# Cognitive remission: a novel objective for the treatment of major depression?

**DOI:** 10.1186/s12916-016-0560-3

**Published:** 2016-01-22

**Authors:** Beatrice Bortolato, Kamilla W. Miskowiak, Cristiano A. Köhler, Michael Maes, Brisa S. Fernandes, Michael Berk, André F. Carvalho

**Affiliations:** Department of Neurosciences, University of Padova, Padova, Italy; Psychiatric Centre Copenhagen, Copenhagen University Hospital, Rigshospitalet, Copenhagen, Denmark; Translational Psychiatry Research Group and Department of Clinical Medicine, Faculty Medicine, Federal University of Ceará, Rua Prof. Costa Mendes 1608, 4° andar, Fortaleza, CE 60430-040 Brazil; Deakin University, School of Medicine, IMPACT Strategic Research Centre, Geelong, Australia; Department of Psychiatry, Faculty of Medicine, Chulalongkorn University, Bangkok, Thailand; Laboratory of Calcium Binding Proteins in the Central Nervous System, Department of Biochemistry, Federal University of Rio Grande do Sul, UFRGS, Porto Alegre, Brazil; Orygen, The National Centre of Excellence in Youth Mental Health, Department of Psychiatry and The Florey Institute of Neuroscience and Mental Health, University of Melbourne, Parkville, Australia

**Keywords:** Antidepressants, Cognition, Cognitive enhancers, Erythropoietin, Lisdexamfetamine dimesylate, Major depression, Novel targets, Vortioxetine, Psychiatry

## Abstract

**Background:**

Cognitive dysfunction in major depressive disorder (MDD) encompasses several domains, including but not limited to executive function, verbal memory, and attention. Furthermore, cognitive dysfunction is a frequent residual manifestation in depression and may persist during the remitted phase. Cognitive deficits may also impede functional recovery, including workforce performance, in patients with MDD. The overarching aims of this opinion article are to critically evaluate the effects of available antidepressants as well as novel therapeutic targets on neurocognitive dysfunction in MDD.

**Discussion:**

Conventional antidepressant drugs mitigate cognitive dysfunction in some people with MDD. However, a significant proportion of MDD patients continue to experience significant cognitive impairment. Two multicenter randomized controlled trials (RCTs) reported that vortioxetine, a multimodal antidepressant, has significant precognitive effects in MDD unrelated to mood improvement. Lisdexamfetamine dimesylate was shown to alleviate executive dysfunction in an RCT of adults after full or partial remission of MDD. Preliminary evidence also indicates that erythropoietin may alleviate cognitive dysfunction in MDD. Several other novel agents may be repurposed as cognitive enhancers for MDD treatment, including minocycline, insulin, antidiabetic agents, angiotensin-converting enzyme inhibitors, S-adenosyl methionine, acetyl-L-carnitine, alpha lipoic acid, omega-3 fatty acids, melatonin, modafinil, galantamine, scopolamine, N-acetylcysteine, curcumin, statins, and coenzyme Q10.

**Summary:**

The management of cognitive dysfunction remains an unmet need in the treatment of MDD. However, it is hoped that the development of novel therapeutic targets will contribute to ‘cognitive remission’, which may aid functional recovery in MDD.

## Background

Major depressive disorder (MDD) is a prevalent and disabling condition with a high frequency of non-recovery and recurrence, leading to substantial mortality and morbidity [1–3]. Only a subset of individuals with MDD (30–40 %) reach symptomatic remission after adequate treatment with a first-line antidepressant, and many patients do not reach premorbid levels of psychosocial functioning, such that a significant proportion of patients present residual symptoms. Cognitive symptoms are acknowledged clinical manifestations of MDD, with fatigue, sleepiness, and executive dysfunction being among the most frequently reported residual manifestations of MDD [4]. The ICD-10 and DSM-5 diagnostic criteria consider items such as ‘reduced concentration and attention’ and ‘diminished ability to think or concentrate’, respectively, in relation to MDD.

On average, the standardized effect size of cognitive impairment in a first major depressive episode (MDE) compared to that in healthy controls is approximately 0.3. Accordingly, a meta-analysis of 13 studies in 644 symptomatic patients with first-episode depression versus 570 healthy controls showed significant impairments in psychomotor speed, attention, and most aspects of executive function (i.e. attentional switching, verbal fluency, cognitive flexibility), with small to moderate effect sizes [5]. However, a subpopulation of MDD patients (approximately 20 %) exhibited deficits of two standard deviations below the mean on two or more cognitive domains [6]. Notwithstanding the heterogeneity across individual studies, the most replicated findings indicate that cognitive impairment in MDD encompasses several domains, notably attention, memory, psychomotor speed, and executive function [7–9]. Impairments in executive function and attention may persist after the affective symptoms are fully mitigated [10]. In addition, a significant proportion of patients with MDD do not recover baseline levels of psychosocial functioning even when they meet the conventional definition of remission (e.g. a score ≤7 on the Hamilton Depression Rating Scale (HDRS)) [2, 11, 12], suggesting that non-affective dimensions and domains are critical determinants of functional outcome. This finding is particularly important when considering that impairments in psychosocial functioning, notably decrements in work productivity, are key determinants of MDD-related societal costs [13]. Several lines of evidence suggest that cognitive dysfunction is an important mediator of functioning, including workforce performance, in MDD [14–16].

Despite standard antidepressants, many individuals continue to complain of and/or manifest cognitive deficits contributing to a diminished quality of life and functional outcome [17–20]. Furthermore, some pilot studies show that cognitive deficits in MDD patients may predict non-response to treatment with selective serotonin reuptake inhibitors (SSRIs) or dual serotonergic-noradrenergic reuptake inhibitors (SNRIs), indicating that poor performance in selected cognitive domains may define a patient subgroup that requires complex or additional treatment [21–23].

The aim of this opinion article is to summarize evidence of possible procognitive effects of standard antidepressant agents and to describe novel therapeutic targets for the management of neurocognitive impairment in MDD. We briefly review relevant clinical aspects pertaining to the assessment and effect of cognitive impairment in adults with MDD since we have critically evaluated these in a previous article [24].

## Discussion

A significant body of evidence shows that individuals with MDD exhibit clinically significant cognitive impairment across multiple domains. The most frequent findings include small to moderate deficits in attention [22], working memory [25], learning [10], processing speed [26], and executive functions [27].

### Methodological challenges in the study of cognition in MDD

Insight into the nature, course, and implications of cognitive dysfunction in MDD has been limited by heterogeneous demographic features (e.g. age and educational attainment), clinical compositions (e.g. first, multi-episode or chronic depression, severity of symptoms, depression subtypes, age of onset, substance use, and comorbid medical conditions), and treatment assignments. Altogether, these variables likely play a major role in producing the inconclusive findings reported in available studies [28]. In addition, the lack of gold-standard tools to assess cognition in MDD and the variety of neuropsychological tests employed in different investigations hinder clear comparisons between studies. Moreover, proper adjustment for confounding factors (e.g. depression severity or practice effects) is frequently lacking, which further complicates the analysis of available evidence [29]. A further limitation of these approaches for evaluating cognitive dysfunction is their comparison with healthy, age-matched control groups. Cognitive dysfunction on neuropsychological tests may thus not be detectable in patients with supra-normal premorbid function, despite their cognitive decline after onset of illness (which may be evident in their subjective cognitive function). Nevertheless, these patients could still be significantly disabled because of the functional impact of their cognitive decline in work settings [30], particularly if their job involves complex planning and fast decision making. Therefore, evaluation of objective neuropsychological function should ideally be accompanied by an assessment of subjective cognitive function. Notably, however, subjective and objective neuropsychological measures of cognition are not closely related [31, 32], indicating that patients with the greatest objective cognitive dysfunction do not necessarily experience the most pronounced cognitive problems, and vice versa. This finding highlights a role of other factors (e.g. depressive symptoms) in the poor correlation between objective and subjective cognition measures [31, 32]. Indeed, there are several important methodological challenges in the study of cognition in MDD that must be addressed in future studies.

### Meta-analytic evidence for non-specific cognitive dysfunction

Notwithstanding these limitations, separate meta-analyses provide compelling evidence that (objective) cognitive impairment is a consistent, replicable, non-specific, and clinically significant feature of MDD – and, for that matter, many major psychiatric disorders [10, 14, 33–35]. For instance, a meta-analysis of 24 studies in which all participants had been assessed with the same comprehensive neuropsychological battery (Cambridge Neuropsychological Test Automated Battery) showed significant impairments in executive functions, attention, and memory in the symptomatic phases of MDD (Cohen’s d effect sizes ranging from −0.34 to −0.65) [10]. In keeping with the view that broad-based cognitive deficits are associated with MDD, the presence of significant deficits in executive functions and psychomotor speed in adults with MDD has been confirmed by a separate meta-analysis of 113 studies (Cohen’s d effect sizes ranging from −0.32 to −0.97) [34].

### Course of cognitive dysfunction in MDD

Cognitive dysfunction in depression does not appear to be simply an epiphenomenon of mood symptoms (e.g. fatigue) but instead is a core and persisting phenomenon [36]. For example, a systematic review of 11 studies involving 500 adult patients in remission from MDD documented persistent deficits in sustained and selective attention, memory, and executive function. However, the heterogeneity across studies did not allow for the estimation of precise effect sizes [37]. In a subsequent study of individuals with unipolar depressive disorder in remission, Hasselbalch et al. [38] reported that the severity of cognitive impairment increases as a function of the cumulative duration of MDEs and, additionally, indicated that the occurrence of psychotic features is associated with worse cognitive performance. Finally, a meta-analysis of 27 studies investigating euthymic adult subjects with MDD showed that executive dysfunction (namely lower inhibitory control) could persist after improvement of depressive symptoms [39]. Moreover, these analyses noted an effect of age as a correlate of poor cognitive performance in MDD, introducing disparate etiopathogenic mechanisms (e.g. vascular and neurodegenerative factors).

Notwithstanding the correlation between episode frequency and deficits in cognitive performance, clinically significant deficits in cognitive function have been reported among adults with MDD experiencing an index depressive episode. For example, a meta-analysis of 13 studies on adults with a first MDE reported significant impairment in psychomotor speed (effect size 0.48), attention (effect size 0.36), and visual learning and memory (effect size 0.53) compared with healthy controls [5]. In addition, a recent meta-analysis of 17 studies comparing 447 children and adolescents with MDD with 1,347 healthy controls indicated significant deficits in inhibitory control, phonemic verbal fluency performance, verbal memory, sustained attention, and planning ability, as well as in working memory and shifting ability (random effects standardized mean difference ranging from 0.21 to 0.77) among MDD patients [40].

Furthermore, preliminary evidence suggests that cognitive impairment may even pre-date the onset of clinically significant depressive symptoms. Different population-based studies have suggested that poor performance in tests of episodic memory may predict the onset of depressive symptoms [26, 41]. Moreover, the large-scale Vietnam Era Twin Study showed that, after adjustment for confounders, a lower score on a general cognitive measure was associated with increased risk of developing depressive symptoms [42]. Finally, this assumption was further supported by the findings of a Danish study of high-risk twins [43].

Taken together, using the classification proposed by Davis et al*.* [44], these studies suggest that cognitive impairment may represent a risk biomarker that may even antedate illness onset, a trait marker present in remitted patients, a marker of progression showing greater severity with illness progression, and a state or acuity marker of MDD, with more marked effects on acutely ill individuals. In addition, cognitive impairment is a principal determinant of quality of life and function. Thus, it could be postulated that aspects of cognitive dysfunction in MDD may reflect a variety of divergent processes in this illness.

### Clinical implications of cognitive dysfunction in MDD

Current evidence supports a putative mediational role of cognitive dysfunction in psychosocial functioning, notably workforce productivity [45, 46]. Importantly, it has been suggested that workplace impairment may contribute to more than 60 % of the MDD-related economic burden [47].

Data from The European Study of the Epidemiology of Mental Disorders, a cross-sectional survey including 21,425 adults from six European countries, reported that cognitive deficits and embarrassment (i.e. stigma) account for half of the association between a MDE and work loss [15]. Consistent with these data, results from a study involving fully employed adults with MDD documented a significant interference of subjective cognitive deficits with workplace role-functioning, regardless of antidepressant therapy [48]. In this same vein, Jaeger et al. [16] measured the neurocognitive performance of patients following hospitalization for an MDE and documented that more pronounced neurocognitive deficits at 6 months follow-up were associated with poorer functional outcome and greater disability. A recent systematic review further suggests a putative mediational role of neurocognitive impairment in functioning, including workforce performance, among individuals with MDD [30].

In recent decades, the target clinical endpoints for MDD treatment have evolved from response (i.e. a 50 % reduction in severity of depressive symptoms from baseline) to the objective of clinical remission [49]. In research settings, the definition of remission is based on the achievement of specific cut-off scores on rating scales of depressive symptom severity (e.g. 17-item HDRS score ≤7; Montgomery-Asberg Depression Rating Scale (MADRS) score ≤10) [50, 51]. However, the concept of remission provides only a vague theoretical definition that may be influenced by the psychometric limitations of available instruments [52, 53]. Even a score of 7 on the HDRS, for example, may not reflect true remission [54], and it has been suggested that lower scores (<5) correlate with objective cutoff points. Furthermore, remission does not equate to recovery. In fact, the acknowledgement that even subthreshold depressive symptoms may be associated with substantial psychosocial impairment has led some experts to postulate that functional recovery is likely to represent the proper target for MDD treatment [2]. Improvements in quality of life are important for long-term social functioning and have been increasingly considered targets for MDD treatment. However, quality of life measures are significantly influenced by age, depressive symptom severity, and episode recurrence, as well as chronic somatic or painful comorbidities and treatment status (e.g. number of medications or antidepressant switch) [55, 56]. Since cognitive dysfunction is one of the residual symptoms of MDD that most strongly impairs quality of life and since accumulating evidence suggests that persistent cognitive impairment prevents full recovery even following the resolution of depressive episodes, some authors have advocated for the achievement of ‘cognitive remission’ as an appropriate, novel aim for MDD treatment [57, 58].

### Cognitive effects of conventional antidepressants

#### Direct or indirect effects on cognition?

A number of clinical studies have primarily evaluated the effect of conventional antidepressants on cognitive performance in individuals with MDD, although most of these studies have not evaluated cognitive function as a primary outcome. In addition, the lack of a placebo control and proper statistical control for the effects of clinical severity (i.e. through path analysis) may contribute to inconsistencies across findings. Nevertheless, conventional antidepressants are generally associated with a beneficial effect on cognitive impairment in individuals with MDD, which may be mediated at least in part by improvement in affective symptoms. For example, it was reported that SSRI treatment led to a significant improvement in memory performance (i.e. immediate and delayed verbal, immediate visual, and declarative memory) in individuals with MDD [59, 60]. Moreover, two studies showed that treatment with sertraline is associated with significant improvements in psychomotor speed and executive functions [61, 62]. On the other hand, other findings indicated that sertraline, citalopram, or paroxetine administration, were not associated with significant changes in cognitive performance in a cohort of elderly patients with major depression, whereas a beneficial effect was observed in patients with minor depression, regardless of changes in the severity of depressive symptoms and concomitant benzodiazepine or psychotropic drug use [63].

In a recent 8-week, double-blind, comparative randomized controlled trial (RCT) of SSRIs (escitalopram) and bupropion on adults with MDD (n = 36), both treatments were significantly associated with improved immediate as well as delayed verbal and non-verbal memory, global function, and measures of work productivity [64]. Moreover, no significant between-group differences were observed in this study, in contrast to data obtained from a cross-sectional naturalistic study that reported a superior effect of bupropion over SSRIs or venlafaxine on cognitive function [65]. However, whereas in this study the SSRI group (n = 27) performed significantly worse than healthy controls in tests of psychomotor and processing speed and cognitive flexibility, and the venlafaxine group performed worse than controls in measures of processing speed, the bupropion group (n = 27) did not differ from healthy controls in any cognitive measure; this aspect limits the generalizability of these findings.

The beneficial effects of mirtazapine on cognition after 3 months of treatment were reported in a study of individuals in an acute MDE [66]. Importantly, improvements in cognitive performance were not correlated with the degree of amelioration of depressive symptoms, suggesting that they were not entirely accounted for by the resolution of the MDE. Finally, tianepine treatment for 3 months significantly improved cognitive functions (i.e. short-term memory and learning, as well as reaction time and attention) in patients with mild and moderate MDD [67]. These designs are, however, unable to disentangle indirect effects related to the resolution of depressive symptoms from direct pro-cognitive effects.

Interventions that target multiple neurochemical systems simultaneously (e.g. SNRIs) might be more likely to improve cognitive performance than treatments targeting only a single system (e.g. SSRIs) [68]. For instance, a trial including adults with MDD treated with escitalopram (SSRI; n = 36) or duloxetine (SNRI; n = 37) for 24 weeks showed that SNRIs were superior to SSRIs in ameliorating memory performance [19]. Despite both escitalopram and duloxetine improving measures of working memory, attention, executive function, mental processing and speed, and motor performance, the improvement induced by duloxetine was greater than that induced by escitalopram in episodic and working memory. A caveat, however, is the assessment of cognition as a secondary outcome [19]. Nevertheless, another double-blind RCT investigated the effect of duloxetine treatment on cognition deficits in elderly subjects with recurrent depressive episodes as a primary outcome [69]. Duloxetine treatment for 8 weeks was associated with an increase in a composite cognitive index relative to that measured for a placebo group. This global improvement was indexed by increases in measures of verbal learning and recall, along with greater decreases in depression severity (assessed by HDRS and the Geriatric Depression Scale). Changes in the cognitive composite index were partially independent of improvements in overall depression severity. More recently, the cognitive-enhancing effects of duloxetine have been further supported by a 12-week study including adults presenting MDD with baseline cognitive deficits (n = 30) [70]. Path analysis showed that better cognitive performance was determined not only by decreases in depression severity but also by enhancements of verbal learning and memory performance. However, inconsistent with these findings, duloxetine did not differ from a placebo in several assessed cognitive domains in a recent study on elderly individuals with MDD [71].

A recent meta-analysis further indicates that standard antidepressant drugs may ameliorate certain cognitive domains in patients with MDD, namely psychomotor speed and delayed recall [20]. However, the evidence base remains limited, and the effect sizes derived from this meta-analysis were small in magnitude (standard mean difference was 0.16 for psychomotor speed and 0.24 for delayed recall, respectively). In addition, drawing firm conclusions on the comparability of procognitive effects of antidepressants with distinct mechanisms of action requires further well-designed studies. Evidence of direct procognitive effects of antidepressants is provided by studies in which cognitive dysfunction is specified as a primary outcome of interest, along with the inclusion of a placebo group and appropriate statistical approaches, such as path analysis, to parse direct effects from more pseudo-specific effects such as cognitive changes secondary to mood improvement. Similarly, a recent systematic review indicates that although augmentation therapy may potentially improve cognitive performance after remission of a MDE, available evidence should be interpreted cautiously because of significant heterogeneity in study design and treatment duration across studies [72].

#### Cognition as a predictor of antidepressant efficacy

Few studies have investigated whether neuropsychological measures predict response to treatment with antidepressants. For instance, a small study that enrolled adults with MDD (n = 20) evaluated the effects of 8 weeks of bupropion treatment (150 mg/day) on cognitive performance and reported that lower pretreatment performance on visual memory and processing speed tasks predicts better treatment response [23], supporting the hypothesis that depressed patients with psychomotor slowing may show greater response to bupropion. Conversely, a neurocognitive profile characterized by psychomotor slowing may aid in the identification of a subgroup of patients with MDD who are less likely to respond to SSRI monotherapy [73]. For instance, a study on 70 adult individuals with MDD and 57 healthy controls assessed the value of certain neurocognitive tests of executive functions and psychomotor speed as differential predictors of response to SSRIs (escitalopram or citalopram), dual therapy, or bupropion [74]. Whereas poor pretreatment word fluency performance predicted non-response to SSRIs and longer choice reaction time on the Stroop Test predicted non-response to SSRIs or dual therapy, patients who responded to bupropion tended to show the opposite pattern of neurocognitive performance, exhibiting slower reaction time and poorer pretreatment word fluency performance compared with those of controls. Thus, the authors posit that certain neurocognitive tests, mostly those that depend on psychomotor speed, may serve as differential predictors of clinical response to an SSRI or to an SNRI as opposed to bupropion.

In adults with late-life MDD (n = 177), greater pretreatment verbal memory was associated with better treatment response [75]. Conversely, in elderly subjects (age >65 years; n = 58) with MDD, executive dysfunction was associated with increased risk of relapse after 16-week therapy with nortriptyline, as well as higher recurrence rates after a 2-year placebo-controlled maintenance phase [76]. Subjects with late-onset depression (i.e. >60 years old) are likely to experience increased impairment in verbal memory, processing speed, and executive function with respect to depressed adults [39]. In particular, executive deficits in the elderly were found to correlate with increased residual symptomatology, higher recurrence rates, and worse response to antidepressant medication [77, 78]. However, a meta-analysis of 17 studies of late-life MDD indicated that better performance on tests of executive function (i.e. Dementia Rating Scale initiation and perseveration subtest) and non-executive function (including two tests of psychomotor speed, one test of construction, and one test of memory) is associated with response to antidepressant treatment [79]. Thus, the authors concluded that specific cognitive deficits per se do not offer a reliable basis for making clinical judgments about probable illness course, in contrast to the depression-executive dysfunction model, according to which the occurrence of executive dysfunction portends poor response to antidepressant medication.

#### The confounding effect of age

The major caveat of studies showing the efficacy of antidepressant therapy in treating cognitive deficits is the over-representation of elderly subjects (age >65 years) in the samples, which limits their external validity. Indeed, the effects of antidepressants on cognitive performance in the elderly are likely negatively influenced by the combined effects of comorbidities and a greater likelihood of side effects. Notwithstanding the paucity of data showing beneficial effects of antidepressants on cognitive performance in non-elderly adult subjects with MDD, converging evidence supports the assumption that the clinical magnitude of antidepressant effects is also small in this population. A systematic review showed that different antidepressant treatments induced smaller changes in measures of executive function and attention, despite clinically perceived changes in verbal learning, memory, verbal fluency, and psychomotor speed. Thus, these factors might represent trait-like markers of MDD [72].

#### Cognitive side effects of antidepressant medication

Long-term therapy with certain antidepressants may also be linked to cognitive side effects in adult subjects with MDD. For instance, treatment with antidepressants has been associated with increased cognitive deficits, such as apathy, inattentiveness, forgetfulness, word-finding difficulty, and mental slowing, in depressed individuals reaching partial or full remission (n = 117) [80]. Significant impairment of executive functioning (i.e. phonemic fluency) has also been reported following treatment with SSRIs [60]. Accordingly, another cross-sectional study that investigated the effects of different antidepressants on cognitive performance suggested that class-specific side effects may account, at least in part, for the long-lasting cognitive deficits observed in subjects with MDD in remission [81]. The study used the Wechsler Memory Scale-Revised and the Stroop Color and Word Test to assess memory and executive function in depressed subjects treated with tricyclic antidepressants (TCA; n = 29), SSRIs/SNRIs (n = 21), or medication free (n = 19), as well as healthy controls (n = 31). Individuals from both antidepressant groups, but not from the medication-free group, showed poorer performance in visual memory with respect to healthy controls. Regarding executive function, only subjects in the TCA group showed worse performance than the controls. This deleterious effect might be explained at least in part by the anticholinergic effects of some TCAs [82]. Despite evidence of cognitive impairment in medication-free individuals with MDD [83], different domains of global cognition might be negatively affected by class-specific antidepressant side effects. The pathways whereby this effect occurs are opaque; changes in appetite and energy balance may have negative effects on cognition because interference in the metabolism of glucose may lead to insulin resistance [84], abnormal neurotransmitter turnover, mitochondrial dysfunction, and energy deficits, as well as changes in neural plasticity [85]. Finally, psychotropic agents may have a similar effect on the immune system and, consequently, on cognitive performance [86, 87].

### Cognitive dysfunction as a target for novel treatments

Despite the recent discovery of compounds with potential therapeutic use in MDD-related cognitive dysfunction, there are still few choices available. However, some compounds under preliminary investigation show promising results with respect to their procognitive potential. There is also great research interest in the development or repurposing of various agents as cognitive enhancers for MDD. Herein, we discuss putative treatment options to conventional treatments that deserve future research consideration (Table 1).

**Table 1:** Potential therapeutic targets for the treatment of cognitive dysfunction in major depressive disorder (MDD)

Agent	Putative mechanisms of action	Clinical evidence [Ref. No.]
Vortioxetine	5-HT_3_/5HT_7_ receptor antagonist; partial agonist at the 5-HT_1B_ receptor; agonist at 5-HT1A receptor; inhibitor of the 5-HT transporter	Two multicenter RCTs having cognitive performance as the primary outcome measure were conducted in participants with MDD [91, 92]. Overall, vortioxetine displayed a significant procognitive effect over several domains, which was largely independent of the amelioration of affective symptoms. However, a recent meta-analysis found that the overall effect size was small (0.34) [20].
Lisdexamfetamine dimesylate	D-amphetamine prodrug; enhances the efflux of dopamine and norepinephrine in the CNS	A RCT found LDX augmentation to be efficacious in reducing self-reported executive dysfunction among participants with MDD (N = 143) with residual depressive symptoms [94].
Erythropoietin	Readily crosses the BBB and increases the production of BDNF	EPO improved verbal learning and memory in a preliminary RCT involving participants with treatment-resistant MDD (N = 40) [97]. This effect was largely mood-independent. However, cognitive performance was not the primary outcome measure in this trial.
Minocycline	Promotes hippocampal neurogenesis; Anti-apoptotic effects; Anti-inflammatory activity; Antioxidant; Modulates glutamatergic transmission; Stabilizes the microglia	No clinical trial has investigated the potential procognitive effects of minocycline in samples with MDD.
Thiazolidinediones	Antagonist of PPAR-gamma; increased the production of BDNF; has anti-inflammatory and antioxidant activities	No published clinical trial has investigated the effects of thiazolidinediones upon cognition in samples with MDD.
S-adenosyl methionine	Major methyl-donor; essential for the synthesis of several neurotransmitters; involved in the synthesis of glutathione	A post-hoc analysis of a preliminary RCT involving 40 SSRI-resistant participants with MDD found SAMe to improve in self-rated recall and word-finding difficulties compared to placebo [127].
Omega-3 PUFAs	Anti-inflammatory and antioxidant activities; Increases the production of BDNF; diminishes microglia-related neuro-inflammation	No published clinical trials to date have investigated the effects of omega-3 PUFAs on cognitive performance in samples with MDD.
Modafinil	Pleotropic agent that targets several neurotransmitter systems (e.g., 5-HT, GABA, glutamate, orexin, and histamine).	A small open-label trial has found that modafinil augmentation improved executive function in a sample with MDD [147].
Galantamine	Rapidly reversible acetylcholinesterase inhibitor and a potent modulator of the nicotinic receptor; affects monoamines, GABA and glutamate neurotransmitter systems.	Two preliminary RCTs have found no evidence for a procognitive effect of galantamine augmentation in participants with MDD [150, 151].
Scopolamine	Potent muscarinic antagonist; modulates 5-HT, neuropeptide Y, dopaminergic, and glutamatergic systems.	A proof-of-concept RCT did not observe significant effects of scopolamine in a task measuring sustained attention in a sample with MDD [154].
N-acetylcysteine	Pleotropic agent that modulates glutamate transmission; antioxidant; anti-inflammatory effect; anti-apoptotic activity; increases glutathione.	No published trial has investigated the effects of NAC on cognitive function in samples with MDD.
Statins	Increases BDNF; antioxidant; anti-inflammatory; inhibits the enzyme IDO; modulates the microglia.	No published trial has investigated potential procognitive effects of statins in samples with MDD.

#### Vortioxetine

Vortioxetine (Lu AA21004) is an antidepressant that acts as an antagonist of the 5-HT_3_ and 5-HT_7_ serotonin receptors, partial agonist of the 5-HT_1B_ serotonin receptor, agonist of the 5-HT_1A_ receptor, and inhibits the serotonin transporter [88]. Preclinical studies have shown that vortioxetine may influence learning and memory processes by improving hippocampal synaptic plasticity and increasing the output of pyramidal cells [89]. Furthermore, it has been postulated that vortioxetine may improve the cognitive symptoms of MDD through its effects on cognate serotoninergic receptors, which may modulate glutamatergic neurotransmission [90].

Procognitive effects were suggested by a preliminary RCT that investigated vortioxetine treatment in non-demented subjects with recurrent MDD (n = 453) [71]. Although these were secondary outcome measures, vortioxetine-treated individuals showed increased learning and memory performance (assessed by the Rey Auditory Verbal Learning Test; RAVLT) and higher processing speed (assessed by the Digit Symbol Substitution Test; DSST) compared with a placebo group, and the effects were independent of the reduction in depression severity (assessed by HDRS). Furthermore, a large double-blind 8-week-long RCT investigated the effects of vortioxetine (10 mg or 20 mg) on cognitive performance as the primary outcome measure in adults with recurrent, moderate-to-severe MDD [91]. The study showed that vortioxetine significantly increased a composite index calculated from the DSST and the RAVLT scores, as well as self-reported cognitive tests (i.e. Perceived Deficits Questionnaire). Path analyses showed that this effect was largely attributable to a direct effect of treatment on cognition because one-half to two-thirds of the procognitive effect could not be the result of the overall reduction in depression symptoms.

In accord with these findings, vortioxetine procognitive effects were confirmed as a primary outcome in another 8-week duloxetine-referenced RCT in individuals with moderate-to-severe MDE who reported subjective cognitive dysfunction (n = 602). Significant improvements in objective and subjective measures of cognitive function (i.e. Number of Correct Symbols in the DSST and the attention/concentration or planning/organization subscores of the Perceived Deficits Questionnaire) were observed at the endpoint in the group treated with vortioxetine compared with those of a placebo group, whereas duloxetine was superior to the placebo only with respect to the subjective measures of cognitive function. Both vortioxetine and duloxetine were significantly superior to the placebo in reducing depressive symptoms assessed by MADRS. Path analysis showed that, although more than one-half of the duloxetine effect on cognitive deficits was attributable to improvements in depressive symptoms, the effect of vortioxetine on cognitive performance was largely independent of the alleviation of depressive symptoms [92].

#### Lisdexamfetamine dimesylate

Lisdexamfetamine dimesylate (LDX) is a pharmacologically inactive pro-drug of D-amphetamine approved for Attention Deficit Hyperactivity Disorder treatment. In a proof-of-concept RCT on MDD patients exhibiting residual symptoms after treatment with the SSRI escitalopram, add-on LDX administration was effective in reducing depressive symptoms (assessed by MADRS) [93]. The procognitive effects of LDX in MDD were suggested by an RCT that investigated LDX effects on cognitive performance in adult subjects with mild MDD (n = 143) and long-lasting executive impairment as assessed by the Behavior Rating Inventory of Executive Function – Adult Version self-report global executive composite T score. LDX (20–70 mg/day) used as augmentation therapy improved the executive deficits from baseline to endpoint with respect to the placebo group [94]. However, one limitation of this study was the use of a self-report questionnaire to assess executive function since subjective ratings of cognitive deficits do not necessarily correlate with objective impairments [95]. Another RCT with the primary objective of evaluating the effect of adjunctive LDX (20–50 mg/day) on residual symptoms and cognitive impairment in adults partially responsive to SSRIs or SNRIs is currently under way (ClinicalTrials.gov Identifier: NCT01148979).

#### Erythropoietin

Erythropoietin (EPO) is a glycoprotein primarily synthesized by the kidney that acts as a hormone in the regulation of erythropoiesis. EPO crosses the blood–brain barrier and exerts antidepressant-like and neuroprotective effects enhancing hippocampus-dependent memory and neuroplasticity, partly via increased production of brain-derived neurotrophic factor (BDNF) [95]. In healthy volunteers, EPO modulated neuronal activity in a frontoparietal network during working memory and improved executive function (i.e. verbal fluency) 1 week after a single high-dose administration (40,000 IU) [96].

Clinical evidence of EPO procognitive potential has been provided by an RCT involving moderately depressed individuals (n = 40) with treatment-resistant MDD [97]. Although EPO did not show effects distinct from those of a placebo on the primary outcome measure (i.e. HDRS scores), it produced long-term mood-independent improvement in verbal learning and memory on the RAVLT. Notably, these effects were accompanied by (and correlated with) reversal of subfield hippocampal volume loss [98]. Taken together, these findings suggest that EPO may have cognitive-enhancing effects in MDD. However, these preliminary findings require replication in a large-scale RCT.

#### Minocycline

Minocycline is a broad-spectrum tetracycline antibiotic commonly used to treat acne, infections of the respiratory trait, and mild rheumatoid arthritis. Minocycline is the most lipid-soluble among tetracycline antibiotics and shows the greatest penetration into the cerebrospinal fluid and the central nervous system. Minocycline promotes hippocampal neurogenesis and exerts anti-apoptotic and anti-inflammatory activities, reducing the expression of pro-inflammatory cytokines (e.g. IL-1β, IL-6, IL-2, TNF-α, INF-γ) and up-regulating anti-inflammatory cytokines (e.g. IL-10) [99]. Moreover, it exerts antioxidant properties by suppressing free radical generation and attenuates increases in lipid peroxidation [100], modulates glutamate transmission reducing glutamate release and excitotoxicity, and may indirectly target the monoaminergic system [101]. All these processes have been posited to play a role in the neuroprogressive nature of MDD, and there is evidence that pro-inflammatory status may also correlate with poor neurocognitive performance [102].

Preclinical evidence suggests that minocycline may reduce cognitive impairment [103, 104]. However, clinical trials have produced conflicting results. Two separate RCTs on HIV-1-infected individuals with progressive neurocognitive decline reported that treatment with adjunctive minocycline for 24 weeks was not associated with significant improvements in cognitive performance [105, 106]. Nevertheless, add-on administration of minocycline for 22 weeks was associated with improvements in executive functions in individuals with early-phase schizophrenia [107]. Preliminary findings from an open-label study confirmed minocycline antidepressant potential in individuals with unipolar psychotic depression [108]. Further and larger RCT studies investigating minocycline efficacy on depressive symptoms and cognitive dysfunction are warranted.

#### Insulin and antidiabetic agents

Comorbid metabolic disturbances aggravate the burden of depressive illness, in part by sharing common risk factors and pathophysiological pathways, since impaired insulin signaling and insulin resistance have been documented to play an important role in the pathogenesis of MDD [109]. The localization of insulin receptors in the hippocampus and medial temporal cortex suggests that insulin and glucose homeostasis play a role in physiological memory functions through their effect on synaptic plasticity and cellular survival [110]. Insulin exerts anti-apoptotic activities and facilitates neuronal growth and differentiation by promoting synapse formation, thus influencing learning and memory processes. In addition, insulin serves as a neuromodulator by enhancing serotonin synthesis and inhibiting the reuptake of norepinephrine by pre-synaptic neurons. Alterations in insulin signaling are associated with mitochondrial dysfunction and lead to the activation of pro-inflammatory pathways, as well as increased oxidative/nitrosative stress and glutamate excitotoxicity, which result in neuroinflammation and neurodegeneration [111]. Insulin resistance and adiposity have been associated with poor cognitive performance on tasks of attention, memory, and processing speed even in non-diabetic population-based samples of middle-aged individuals [112]. Results obtained from preclinical studies and controlled trials on healthy volunteers and individuals with mild cognitive impairment or Alzheimer’s disease have supported the potential of intranasal insulin in improving memory performance [113]. An RCT among euthymic patients with bipolar disorder documented that individuals treated with intranasal insulin exhibited significant improvements in one measure of executive function (in the absence of improvement across other cognitive domains) compared with a placebo group [114]. An interventional RCT evaluating the putative antidepressant and procognitive effect of intranasal insulin in MDD is currently under way (ClinicalTrials.gov Identifier: NCT00570050).

In keeping with the view outlined above, enhanced insulin signaling with antidiabetic treatments, for example, thiazolidinediones such as rosiglitazone and pioglitazone, may preserve and/or augment cognitive function at least in a subset of MDD patients exhibiting comorbid metabolic conditions [115]. Thiazolidinediones act as agonists of peroxisome proliferator-activated receptor gamma and have been demonstrated to exert neuroprotective effects, reducing oxidative stress and weakening the pro-inflammatory response, as well as promoting neuroplasticity via increased production of BDNF [116]. A pilot, open-label trial involving adult outpatients with MDD (n = 23) with comorbid abdominal obesity (waist circumference >35 inches in women and >40 inches in men) or metabolic syndrome documented that adjunctive pioglitazone significantly improves depressive severity (assessed by the Inventory of Depressive Symptomatology), with a concurrent reduction in insulin resistance and inflammatory markers, as measured by high-sensitive C-reactive protein [117]. In accord, a subsequent 6-week RCT confirmed the antidepressant efficacy of pioglitazone in obese patients with concomitant polycystic ovarian syndrome and MDD (n = 50), reporting significant reductions in HDRS scores [118]. Importantly, the authors of this study underscored that antidepressant properties appeared to be largely independent of insulin-sensitizing action and are possibly mediated by anti-inflammatory and antioxidant actions [118], including the inhibition of the nuclear factor kappa B (NF-κB) cascade and cytokine production, as well as quinolinic acid-induced neurotoxicity [119]. In a 6-week RCT on individuals with moderate-to-severe MDD (n = 40) without diabetes or metabolic syndrome, adjunctive treatment with pioglitazone was associated with greater decrements in HDRS compared with a placebo group, in line with the hypothesis that antidepressant effects are mediated by anti-inflammatory, anti-excitotoxic, and neuroprotective properties [120].

Finally, the anti-diabetic drug metformin holds promise as an agent capable of attenuating and/or reverting neurotoxic effects mediated by inflammatory mechanisms, metabolic and mitochondrial dysfunction, as well as increased oxidative stress [121]. Clinical evidence of beneficial antidepressant and neurocognitive potential was recently documented in an interventional 24-week study testing the effects of metformin relative to those of a placebo in individuals with MDD and comorbid type II diabetes mellitus (n = 58). The effect of metformin was superior to that of placebo in improving depressive symptoms in a sample of depressive patients with type II diabetes mellitus [122]. In addition, metformin improved memory performance (assessed by the Wechsler Memory Scale-Revised), which was significantly related to a decrease in depressive symptoms [122].

#### Angiotensin-converting enzyme (ACE) inhibitors

Hypertension is a risk factor for cognitive impairment as well as for depression. ACE inhibitors have immune-modulating properties and suppress cytokine production (e.g. IL-1, TNF-α), likely through interference with NF-κB activation [123]. Preclinical studies have documented that ACE inhibitors exert memory-improving effects [124]. Preliminary clinical evidence suggests that chronic therapy with these agents in individuals with mild-to-moderate hypertension may improve memory performance, as assessed by the RAVLT and the Wechsler Memory Scale [125]. Thus, this evidence provides proof of principle for direct procognitive effects of ACE inhibitors in humans, and the design of a specific trial aiming to investigate the effects of ACE inhibitors for the remediation of cognitive dysfunction in individuals with MDD is warranted.

#### S-adenosyl methionine (SAMe)

SAMe is formed from the essential amino acid methionine and adenosine triphosphate. SAMe is a major methyl donor required for the synthesis of several neurotransmitters and serves as a precursor molecule to the transsulfuration pathway, which leads to the synthesis of glutathione [126]. The procognitive potential of SAMe was preliminarily documented in a 6-week RCT on 40 SSRI-resistant outpatients with MDD [127]. Participants allocated to SAMe therapy showed significant improvements in recall and word-finding scores on the self-rated Cognitive and Physical Symptoms Questionnaire compared with a placebo group. These promising results deserve replication in an RCT aiming to investigate the possible direct and objective cognitive effects of SAMe as a primary outcome in participants with MDD.

#### Acetyl-L-carnitine (ALC) and alpha lipoic acid (ALA)

ALC is a natural substance involved in both carbohydrate and lipid metabolism and exerts a role in the amelioration of the insulin-resistant state. The pleiotropic actions of ALC include enhanced neuronal metabolism in the mitochondria and a counteracting effect toward glutamate-mediate neurotoxicity and hypothalamic–pituitary–adrenocortical axis hyperactivity. Four separate RCTs suggested the efficacy of ALC over a placebo in mitigating depressive symptoms in MDD [128]. ALC’s beneficial effects on cognition have been documented in the treatment of mild cognitive impairment and Alzheimer’s disease, as well as of hepatic encephalopathy [129, 130]. Taken together, these results provide the rationale for investigating ALC’s efficacy in the treatment of neurocognitive deficits in the context of MDD.

ALA is a cofactor in dehydrogenase complexes that exerts regulatory effects on cellular metabolism. It has been posited that ALA supplementation in MDD would result in antidepressant effects, possibly via increased insulin sensitization and a consequent increase in monoamine synthesis [131]. Moreover, preclinical studies and investigations in individuals affected by Alzheimer’s disease [132] provided evidence that ALA may promote neurocognitive enhancement via pleiotropic mechanisms, including antioxidant mitochondrial activity and anti-inflammatory properties [133], providing a basis for evaluating ALA’s efficacy in MDD as well.

#### Omega-3 polyunsaturated fatty acids (n-3 PUFAs)

n-3 PUFA supplementation may be beneficial in the treatment of several psychiatric disorders, including MDD. Preclinical evidence suggests that a diet enriched in n-3 PUFA (i.e. ethyl-eicosapentaenoic acid) may attenuate IL-1-induced memory impairment and block IL-1-induced increases in serum corticosterone concentration [134]. Moreover, n-3 PUFAs reduce the inflammatory response, limiting the production of pro-inflammatory mediators by microglial cells following ischemic insults, and enhance glutathione-related antioxidant pathways [135, 136]. In middle-aged to elderly healthy individuals (n = 40), n-3 PUFA supplementation (3 mg/day) significantly improved cognitive performance (i.e. working memory and attention) compared with that of a placebo group in a 5-week RCT [137]. In addition, an inverse relationship was observed between cardiometabolic risk factors and cognitive performance, suggesting that dietary prevention strategies may delay the onset of metabolic disorders and associated cognitive decline. Independent clinical trials and meta-analyses of RCTs have also provided evidence of the superiority of n-3 PUFAs, namely eicosapentaenoic and docosahexaenoic acids, over a placebo as augmentation agents in the treatment of MDD [138]. However, few studies have investigated the effects of n-3 PUFAs on cognition, yielding inconclusive results to date. An RCT evaluating the effect of n-3 PUFA (1.5 g/day) supplementation on mood and cognition in mildly to moderately depressed patients did not indicate any significant change in cognitive functions [139]. However, supplementation with n-3 PUFAs in an RCT was effective in improving aspects of emotional information processing in individuals recovered from MDD [140]. Additionally, in a separate cross-sectional investigation on individuals recovered from MDD (n = 132), plasma concentrations of n-3 PUFAs correlated positively with neurocognitive performance [141]. Further investigations are needed to elucidate the effects of n-3 PUFAs on cognitive performance in MDD.

#### Melatonin

Melatonin is a hormone secreted by the pineal gland, which influences the maintenance of circadian rhythms and affects energy metabolism. The mechanisms through which melatonin can exert neuroprotective and cognitive-enhancing effects include anti-inflammatory and antioxidant properties, as well as enhanced neuroplasticity and neurogenesis [142]. In a 6-week double-blind RCT, the combination of melatonin and buspirone provided beneficial effects in treating depressive severity in individuals with MDD [143]. Another RCT investigating 80 individuals with mild-to-moderate Alzheimer’s disease documented that the administration of adjunctive melatonin for 6 months was effective in improving cognitive functions and sleep maintenance [144]. Melatonin, as well as agomelatine, a melatonergic analogue drug acting as an MT_1_/MT_2_ agonist and 5-HT_2C_ antagonist, may hinder circadian misalignment and sleep disruption, which has been posited to contribute to increases in depressive symptoms through their effect on cognitive control [145].

#### Modafinil

Modafinil is a stimulant-like agent currently approved by the US Food and Drug Administration as a treatment for excessive sleepiness in narcolepsy. This drug has a pleiotropic mechanism of action targeting several neurotransmitters, including but not limited to serotonin, GABA, glutamate, orexin, and histaminergic systems [146]. Modafinil has only been tested as a procognitive agent for MDD in a 4-week open-label trial, enrolling 35 patients with MDD with a partial response to an antidepressant at a stable dose [147]. After modafinil augmentation, the authors concluded that no adverse effect on cognition was reported. However, neurocognitive function was not a primary outcome of this small, uncontrolled trial. A recent systematic review indicates that modafinil has procognitive effects in healthy, non-sleep-deprived individuals [148]. Thus, the potential beneficial effects of modafinil in remediating cognitive dysfunction in MDD deserve investigation.

#### Galantamine

Galantamine is a cholinergic agent, a weak acetylcholinesterase inhibitor, and a potent nicotinic receptor modulator. Because of its modulatory effects on nicotinic receptors, it has been proposed that this drug may modulate other neurotransmitter systems, including monoamines, glutamate, and GABA [149]. Two double-blind, RCTs evaluated the effects of galantamine augmentation on neurocognitive function in MDD [150, 151], and both failed to demonstrate beneficial effects on cognition. However, they both enrolled small samples, with that by Holtzheimer et al. [150] enrolling only elderly participants.

#### Scopolamine

Several lines of evidence indicate that supersensitivity of the muscarinic cholinergic system is implicated in the pathophysiology of MDD [152]. Scopolamine, a potent muscarinic antagonist, promotes a rapid and enduring antidepressant effect [153]. In addition to its anticholinergic effect, scopolamine may modulate serotonin, neuropeptide Y, and the dopaminergic and glutamatergic systems. Notwithstanding a preclinical and pharmacological rationale for scopolamine possibly offering procognitive benefits for MDD, results obtained from a small proof-of-concept study failed to provide a sign of efficacy in the cognitive domain (selective attention) [154].

#### N-acetylcysteine (NAC)

NAC is a potentially useful agent for treating a range of psychiatric disorders [155]. NAC acts as a modulator of synaptic glutamate through the cysteine-glutamate exchanger, increases glutathione and oxidative defenses, enhances neurogenesis and mitochondrial function, decreases pro-inflammatory cytokines, and regulates apoptosis [156]. Cognitive studies of NAC have produced inconsistent results, which may be linked to variations in study design [155]. NAC augmentation of standard treatment in Alzheimer’s disease was associated with improvements in verbal abilities and executive control cognitive tasks compared with controls [157]. NAC administration after blast traumatic brain injury was associated with improved executive function compared with that observed for controls [158]. However, NAC pretreatment in healthy subjects with ketamine-induced psychosis did not reduce the effect of ketamine on cognition [159]. In a sample with bipolar disorder, there were no differences between NAC and placebo with respect to cognitive measures [160]. Further, a pooled analysis of participants in two trials of NAC for schizophrenia and bipolar disorder showed better working memory performance in the NAC- compared to the placebo-treated arm (Rapado-Castro M, personal communication). Definitive studies are warranted.

#### Curcumin

Preliminary evidence indicates that add-on treatment with curcumin may promote antidepressant effects in patients with MDD [161, 162]. Evidence also shows that curcumin may improve neurocognitive function. For example, in animal models, administration of curcumin improves chronic, stress-induced, depressive-like behaviors and cognitive defects (e.g. impaired learning and memory) [163]. An RCT examined the acute and chronic effects of curcumin (400 mg/day) on mood and cognitive functions in elderly adults [164]. Acute curcumin administration improved performance on tasks that measured sustained attention and working memory, whereas chronic treatment also improved mood and fatigue [164]. Curcumin is a pleiotropic agent that targets immune-inflammatory and oxidative and nitrosative stress pathways and promotes neuroprotection and improved neurogenesis [165, 166].

#### Statins

Statins have been widely prescribed to lower blood cholesterol and for the prevention of cardiovascular illnesses. Preclinical studies indicate that simvastatin may have anxiolytic and antidepressant-like effects in stress-related murine models [167, 168]. Atorvastatin also promotes antidepressant-like effects and increases hippocampal BDNF levels in rodents [169]. In addition, meta-analytic evidence suggests that statins induce mood-enhancing effects and decrease the risk of development of de novo depression in humans [170, 171]. Recently, some RCTs have tested the efficacy of statins for the treatment of depression. Ghanizadeh and Hedayati [172] randomly allocated 68 participants with MDD to receive either fluoxetine plus placebo or fluoxetine plus lovastatin from the inception of treatment. By the end of this trial, the group allocated to lovastatin augmentation showed a significant reduction in HDRS scores compared with those measured for a placebo group. Another 12-week RCT allocated participants with severe MDD (under standard treatment with citalopram 40 mg/day; n = 60) to receive either adjunctive atorvastatin or a placebo [173]. The group allocated to atorvastatin showed lower HDRS scores by the end of the trial; however, no participant achieved remission. A subsequent 6-week RCT allocated participants meeting criteria for MDD (n = 48) either to fluoxetine plus placebo or to fluoxetine plus simvastatin [174]; at study completion, the latter showed significantly lower depressive symptoms, whereas differences in remission rates did not achieve statistical significance.

Statins may have pleiotropic actions in the central nervous system. For example, simvastatin and atorvastatin inhibit inflammation by decreasing TNF-α and IL-1β levels. Simvastatin was also shown to be neuroprotective in an animal model of ischemia; this effect was associated with an increase in the activity of endothelial nitric oxide synthase. Furthermore, statins may decrease the activity of the enzyme indoleamine 2,3-dioxygenase [175], which is induced by IFN-γ, leading to the cleavage of the essential amino acid tryptophan, a precursor of serotonin and melatonin, as well as the production of neurotoxic tryptophan catabolites [176]. These multiple actions indicate that statins may show promise as procognitive agents for individuals with MDD. However, no trial to date has evaluated the effects of statin members as cognitive enhancers for patients with MDD.

#### Coenzyme Q10 (CoQ10)

CoQ10, also referred to as ubiquinone, is found in all cell types. CoQ10 is a redox modulator, a membrane stabilizer, and a pivotal cofactor of the mitochondrial electron transport chain. As an antioxidant, CoQ10 protects cells from the damaging effects of reactive oxygen species and reactive nitrogen species [177]. CoQ10 also has anti-inflammatory effects via the suppression of the activation of NF-κB and through the modulation of the transcription of genes governing pro-inflammatory JAK/STAT pathways [178]. Furthermore, CoQ10 offers neuroprotection in animal models of neurodegenerative diseases (e.g. Parkinson’s) [179, 180].

CoQ10 has been implicated in the pathophysiology of neuro-immune diseases, including MDD [177]. For example, decreased peripheral levels of CoQ10 have been reported in patients with MDD relative to those measured for controls [177]. CoQ10 displays antidepressant-like effects in chronically stressed rats, with a reduction in hippocampal oxidative/nitrosative DNA damage [181]. These pleiotropic effects indicate that CoQ10 may hold promise as a cognitive enhancer for neuro-immune diseases in which mitochondrial dysfunction is a pathophysiological hallmark, including but not limited to MDD [182].

### Methodological considerations in cognition trials

The results of the above-reviewed studies of novel candidate treatments for cognitive dysfunction in MDD remain preliminary. The few positive results deserve replication in well-designed RCTs involving cognitive dysfunction as a primary outcome in participants with MDD. One key issue is the lack of consensus on whether and how to screen for cognitive dysfunction in these trials. For example, the allocation of ‘cognitively intact’ patients who show no measurable neuropsychological deficits despite their depression-associated subjective cognitive complaints [31, 32], which may introduce ceiling effects, remains problematic. Indeed, in a recent study, we demonstrated that patients with cognitive impairment at baseline had substantially greater chances of achieving a clinically meaningful cognitive improvement in response to EPO treatment compared with patients with normal baseline cognition (Miskowiak et al., unpublished observations). This finding suggests that objective screening for cognitive impairment should be performed in future cognition trials. Another issue is the lack of guidelines on which cognition measures should be implemented as primary outcomes in these trials (i.e. single neuropsychological test measures or composite scores of tests assessing several cognitive domains). Other unresolved issue is whether patients should be in remission or can have current mood symptoms at the time of enrollment. In addition, the allowance of concomitant medications with direct effects on cognition (e.g. benzodiazepines) deserve careful consideration. Moreover, the use of proper statistical methods that aid in the discrimination of direct procognitive effects versus beneficial effects on cognition that occur as a result of mood improvement should also be considered (e.g. path analyses). The lack of consensus regarding these methodological issues is a substantial limitation that must be addressed to advance the field.

## Summary

This review provides a comprehensive evaluation of approved antidepressants and novel therapeutic targets for cognitive dysfunction in major depressive disorder. Although inconsistent but positive effects of conventional treatments on cognitive impairment are observed, there is an absence of compelling evidence of a specific and direct effect with most antidepressants. Among agents within a class of antidepressants, it is also uncertain whether efficacy can be generalized to all members. Furthermore, a major limitation of available evidence is that cognitive improvement was not a primary outcome in most trials. Nevertheless, no single antidepressant agent has received approval by the US Food and Drug Administration for the treatment of cognitive disturbances associated with MDD [72]. A shift towards cognitive function as a priority therapeutic target is occurring in psychiatry across the developmental trajectory [85, 183]. Advances in cognitive neuroscience are providing insight into the neurobiology subserving this domain; it is hoped that the accumulating knowledge might provide the foundation to develop genuinely novel therapeutic approaches.

This review covered several novel therapeutic targets for cognitive impairment in MDD. The data are preliminary across these agents and are promising for EPO, SAMe, insulin, NAC, and antidiabetic agents. Several mechanisms may contribute to cognitive dysfunction in MDD, including but not limited to an over-activation of the hypothalamic–pituitary–adrenal axis, oxidative and nitrosative stress, imbalances in pathways involved in cell survival and death, and immune activation [11]. These pathways may offer a foundation for the development of novel drugs and/or the repurposing of existing agents (e.g. minocycline) for the treatment of cognitive impairment in depression [99]. Furthermore, in light of evidence suggesting that the neurobiological underpinnings of cognitive functions share both overlapping and discrete features with those subserving mood regulation, translational avenues of research are broadening the range of possible pharmacological novel targets relevant to cognitive dysfunction in MDD, including membrane receptors (e.g. metabotropic glutamate receptor 2/3 antagonists) and intracellular pathways, such as protein kinases, phosphodiesterases, and epigenetic modifications [85, 184].

Although not the primary focus of this review, various non-pharmacological treatments that are under active investigation for their putative cognitive-enhancing effects are worth mentioning. For example, non-invasive brain stimulation techniques, namely transcranial magnetic stimulation [185] and transcranial direct current stimulation [186, 187], are promising techniques under investigation for both their antidepressant and cognitive-enhancing potential in MDD [188]. Other avenues of research indicate that psychological interventions might both directly and indirectly ameliorate cognitive performance in individuals with MDD. For example, data from studies evaluating the impact of cognitive remediation therapy on cognitive performance in MDD indicate a positive effect on neuropsychological tasks of attention, verbal learning and memory, psychomotor speed, and executive function [189, 190]. However, the evidence base for psychological treatments for cognitive dysfunction in MDD also remains rather limited [191]. It appears possible that an integrated approach including pharmacological and non-pharmacological treatments could lead to better effects on cognitive dysfunction in MDD.

In conclusion, the achievement of ‘cognitive remission’ remains a distinctive unmet need in the treatment of MDD. Future large-scale controlled trials involving the cognitive symptoms of depression as primary outcomes hold promise and may ultimately change the landscape of care. Furthermore, cognitive deficits should be assessed objectively (i.e. through neuropsychological batteries) and through self-reported measures simultaneously. Several promising novel targets merit investigation as cognitive enhancers for MDD.

## Abbreviations

ACE: Angiotensin converting enzyme
ALA: Alpha lipoic acid
ALC: Acetyl-L-carnitine
BDNF: Brain-derived neurotrophic factor
CoQ10: Coenzyme Q10
DSST: Digit Symbol Substitution Test
EPO: Erythropoietin
HDRS: Hamilton Depression Rating Scale
JAK: Janus kinase
LDX: Lisdexamfetamine dimesylate
MADRS: Montgomery-Asberg Depression Rating Scale
MDD: Major depressive disorder
MDE: Major depressive episode
n-3 PUFA: Omega-3 polyunsaturated fatty acid
NAC: N-Acetylcysteine
NF-κB: Nuclear factor kappa B
RAVLT: Rey Auditory Verbal Learning Test
RCT: Randomized controlled trial
SAMe: S-Adenosyl methionine
SNRI: Serotonergic-noradrenergic reuptake inhibitor
SSRI: Selective serotonin reuptake inhibitor
TCA: Tricyclic antidepressant
